# More Improved Postoperative Range‐of‐Motion Retention After Arthroscopic Lysis of Adhesions After Total Knee Arthroplasty Is Associated With Arthroscopic Fellowship‐Trained Compared With Nonfellowship‐Trained Surgeons

**DOI:** 10.1002/ars2.70088

**Published:** 2026-09-30

**Authors:** George Bcharah, Alexandra Cancio‐Bello, Calvin Nguyen, Eugenia Lin, Mario Hevesi, Cedric J. Ortiguera, Anikar Chhabra, Joshua Bingham

**Affiliations:** ^1^ Department of Orthopaedic Surgery Mayo Clinic Phoenix Arizona U.S.A.; ^2^ Department of Orthopaedic Surgery Mayo Clinic Rochester Minnesota U.S.A.; ^3^ Department of Orthopaedic Surgery Mayo Clinic Jacksonville Florida U.S.A.

## Abstract

**Purpose:**

To describe whether subspecialty training, specifically arthroscopic fellowship training, influences intraoperative range‐of‐motion (ROM) gains, postoperative ROM retention, and lysis of adhesions (LOA) complication rates.

**Methods:**

Between August 1989 and May 2025, all patients who underwent arthroscopic LOA for stiffness after primary total knee arthroplasty (TKA) at a single multisite academic institution with a minimum follow‐up of 3 months were reviewed. Cases were stratified by the surgeon's training: 1 group with arthroscopic fellowship training and another with nonarthroscopic or no fellowship training. Primary outcomes were intraoperative ROM change and postoperative ROM retention compared with intraoperative and pre‐TKA baseline values. Revision and periprosthetic joint infection rates were also collected.

**Results:**

A total of 94 LOA cases were identified, where 48 (51.1%) were performed by nonarthroscopically trained surgeons. Intraoperative ROM improvements were similar between groups in both knee flexion (35.2° arthroscopic vs 31.1° nonarthroscopic, *P* = .130) and extension (2.43° vs 3.91°, *P* = .079). Compared with the intraoperative ROM, procedures performed by arthroscopically trained surgeons showed significantly better long‐term retention of both flexion (loss of 4.85° vs 15.98°, *P *= .006) and extension (gain of 0.50° vs loss of 1.58°, *P* = .036) at postoperative follow‐up. When compared with preprimary TKA baseline ROM, flexion at follow‐up was slightly improved in the arthroscopic group but declined significantly in the nonarthroscopic group (gain of 1.66° vs loss of 21.76°, *P* < .001). Eight revision TKAs (8.51%) occurred after LOA, 6 of which involved nonarthroscopic surgeons, and 1 periprosthetic joint infection case was reported.

**Conclusions:**

LOA performed by arthroscopic fellowship‐trained surgeons results in significantly better retention of postoperative ROM than nonfellowship‐trained surgeons when used for arthrofibrosis after TKA.

**Level of Evidence:**

Level III, retrospective comparative study.

## Graphical abstract

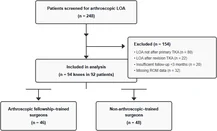

Total knee arthroplasty (TKA) alleviates pain and restores function in most patients, yet postoperative arthrofibrosis remains a notable complication.^1^ Contemporary series report clinically significant stiffness in 6% to 13% of primary TKAs, a problem that drives additional rehabilitation, interventions, and health care costs.^2^, ^3^ Loss of motion after TKA correlates with worse patient‐reported outcomes, difficulty performing activities of daily living, and, in severe cases, early revision.^4^, ^5^, ^6^

When physical therapy fails, manipulation under anesthesia (MUA) and, when indicated, arthroscopic lysis of adhesions (LOA) are the mainstay operative options.^7^ Systematic reviews and recent cohort studies reveal that LOA, often combined with MUA, can augment knee range of motion (ROM) with low complication rates.^7^, ^8^ Nevertheless, the durability of these intraoperative gains is inconsistent. Up to one‐third of patients experience progressive loss of flexion within the first postoperative year, suggesting that factors beyond simple adhesion release, such as surgical technique, may influence long‐term success.^8^

Surgeon‐specific characteristics have emerged as important determinants of outcome across orthopaedic subspecialties. High‐volume arthroscopic surgeons and fellowship‐trained sports medicine surgeons report lower reoperation and emergency department visit rates after arthroscopic rotator cuff repair and anterior cruciate ligament reconstruction.^9^, ^10^ Similarly, recent registry analyses link fellowship training type to reduced complications after primary and revision TKA.^11^ Despite the technical demands of navigating a prosthetic joint arthroscopically, whether dedicated arthroscopic fellowship training confers an advantage during LOA for post‐TKA stiffness remains poorly understood. Exploring the influence of subspecialty training on LOA outcomes may guide referral patterns and optimize functional recovery for patients with post‐TKA stiffness.

The purpose of this study was to describe whether subspecialty training, specifically arthroscopic fellowship training, influences intraoperative ROM gains, postoperative ROM retention, and LOA complication rates. It was hypothesized that patients treated by surgeons with specific arthroscopic fellowship training would show superior ROM retention and lower complication rates compared with those treated by nonarthroscopically trained surgeons.

## METHODS

### Study Design

A retrospective analysis of patients at a single multisite academic institution was performed between 1989 and 2025. Institutional Review Board approval was obtained before data collection. Inclusion criteria included arthroscopic LOA performed for stiffness after primary TKA and a minimum postoperative follow‐up of 3 months. Exclusion criteria included LOA performed for indications other than post‐TKA stiffness, LOA after revision TKA, insufficient follow‐up (<3 months), and missing ROM data. The indication for LOA was persistent stiffness after primary TKA that was refractory to conservative management including physical therapy, with or without prior MUA. Referral to an arthroscopic versus nonarthroscopic surgeon was based on historical referral patterns and surgeon availability within the practice.

### Data Collection

Data was collected through chart review and included the following variables: patient demographics, date of TKA, date of LOA, the calculated interval in days from TKA to LOA, and whether the surgeon was fellowship‐trained, and, if so, whether the fellowship involved arthroscopic training or another subspecialty. Surgeon fellowship status was verified through institutional credentialing records and publicly available curricula vitae.

Preoperative and intraoperative ROM measurements were recorded by the treating surgeon. Postoperative measurements were extracted primarily from the treating surgeon's clinical documentation to minimize interobserver variability; however, given the multiprovider academic setting, measurements recorded by the clinical care team were used when surgeon‐specific data points were unavailable. Extension and flexion measurements were extracted at 4 time points: preoperatively prior to the index primary TKA, pre‐LOA, immediately postoperatively after LOA, and, at final, most recent recorded follow‐up. From these measurements, primary outcomes were measured and included intraoperative changes in ROM during LOA (as recorded in the operative note), post‐LOA to final follow‐up (i.e., ROM retention), and ROM changes at final follow‐up compared with preprimary TKA baseline. Secondary outcomes included requirements for repeat LOA and/or revision TKA. Timing to both procedures to LOA was recorded.

### Statistical Analysis

Continuous variables were presented as mean ± standard deviation. Group comparisons were done using the independent samples *t*‐test or Mann‐Whitney *U* test for continuous data and *χ* ^2^ or Fisher's exact test for categorical data. A 2‐tailed *P* < .05 defined statistical significance.

Revision‐free prosthesis survival was evaluated with the Kaplan‐Meier product‐limit method. “Time 0” was defined as the date of the index LOA. The event of interest was a subsequent revision TKA for any cause, and knees that had not undergone revision were censored at the date of their most recent clinical follow‐up. Equality of the survivor functions was assessed with the 2‐sided log‐rank test (*α* = 0.05). Analyses were performed with RStudio Version 2022.12.0 (RStudio, Boston, MA).

## RESULTS

### Cohort Characteristics

A total of 94 knees in 92 patients met inclusion criteria (Figure 1). From those, 46 procedures (48.9%) were performed by arthroscopic fellowship‐trained surgeons and 48 (51.1%) by nonarthroscopically trained surgeons. In total, there were 23 orthopaedic surgeons, where 13 of them completed a fellowship in orthopaedic sports medicine. Median time from primary TKA to LOA was 1.29 years (interquartile range 0.52‐4.90). Mean follow‐up after LOA was 4.67 ± 3.69 years (range, 0.3‐12.21 years) for the arthroscopic group and 5.04 ± 5.38 years (range, 0.3‐22.98 years) for the nonarthroscopic group (*P* = .73). Baseline characteristics when separated by arthroscopic training were comparable and outlined in Table 1. Baseline pre‐TKA ROM was similar across both cohorts (extension: 1.69 ± 5.60 vs 2.76 ± 4.71, *P* = .318; flexion: 109.55 ± 25.29 vs 108.00 ± 13.69, *P* = .711). Other ROM measurements across the different recording points (intraoperative, post‐LOA, etc.) are shown in Table 2.

**FIGURE 1:**
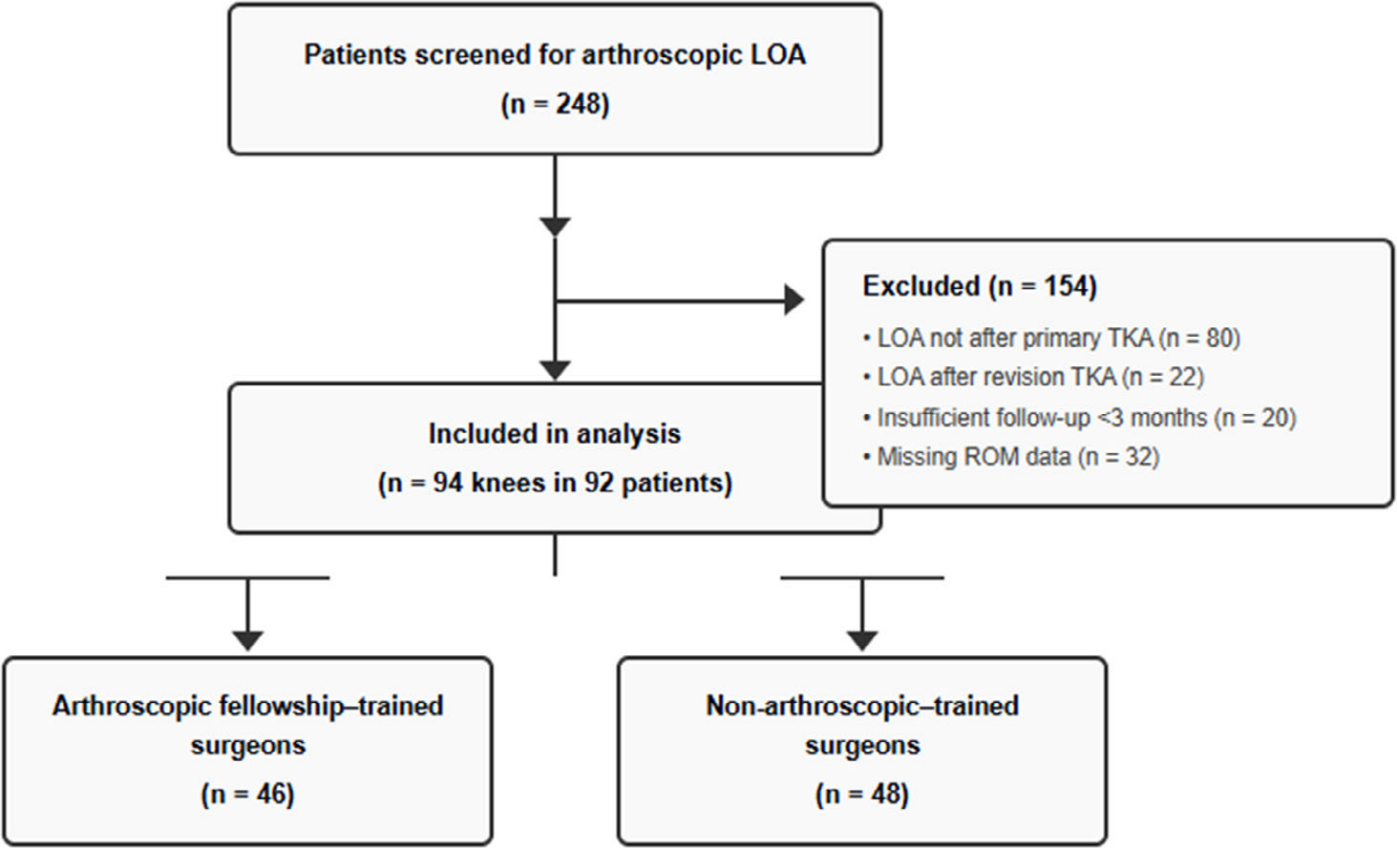
Patient flowchart. CONSORT‐style flow diagram showing patient screening, exclusion criteria, and final cohort allocation by surgeon arthroscopic training status. (LOA, lysis of adhesions; ROM, range of motion; TKA, total knee arthroplasty.)

**TABLE 1:** Baseline Characteristics by Surgeon Arthroscopy Training

Characteristic	Nonarthroscopy Trained (n = 48)	Arthroscopy Trained (n = 46)	*P* Value
Age at procedure	62.72 ± 10.87	59.72 ± 12.57	.193
Women	16 (33.33%)	26 (56.52%)	.093
Race			.368
White	37 (77.08%)	42 (91.30%)	
Black	3 (6.25%)	1 (2.17%)	
Asian	2 (4.17%)	1 (2.17%)	
American Indian/Alaskan Native	0 (0.00%)	1 (2.17%)	
Other	4 (8.33%)	1 (2.17%)	
BMI, mean ± SD	28.30 ± 10.87	31.69 ± 8.44	.132
Anxiety	5 (10.42%)	17 (36.96%)	.009
Fibromyalgia	3 (6.25%)	1 (2.17%)	.594
Myocardial infarction	2 (4.17%)	4 (8.70%)	.693
Congestive heart failure	6 (12.50%)	6 (13.04%)	>.99
Chronic kidney disease	6 (12.50%)	8 (17.39%)	.805
Chronic obstructive pulmonary disease	5 (10.42%)	1 (2.17%)	.196
Diabetes mellitus	11 (22.92%)	16 (34.78%)	.397
Hypertension	13 (27.08%)	32 (69.57%)	<.001
Thyroid disease	5 (10.42%)	13 (28.26%)	.074
Rheumatoid arthritis	8 (16.67%)	3 (6.52%)	.186
Arrhythmia	5 (10.42%)	8 (17.39%)	.578
Cerebrovascular disease	0 (0.00%)	4 (8.70%)	.131
Current smoking	6 (12.50%)	15 (32.61%)	.054

*Note*: Continuous variables are reported as mean ± SD; categorical variables are reported as number (percentage). *P* values were derived using the independent samples *t*‐test for BMI and *χ* ^2^ or Fisher's exact test, as appropriate, for categorical variables.

BMI, body mass index; SD, standard deviation.

**TABLE 2:** ROM Measurements Stratified by Surgeon Arthroscopic Fellowship Training Across Different Time Points Relative to the LOA Procedure

Variable	Nonarthroscopically Trained Surgeons ROM (°), Mean ± SD (n = 48)	Arthroscopic Fellowship‐Trained Surgeons ROM (°), Mean ± SD (n = 46)	*P* Value
Preprimary TKA			
Extension	2.76 ± 4.71	1.69 ± 5.60	.318
Flexion	108.00 ± 13.69	109.55 ± 25.29	.711
Pre‐LOA			
Extension	8.07 ± 9.11	2.52 ± 6.22	.001
Flexion	69.65 ± 20.38	82.85 ± 20.52	.002
Immediate postoperative LOA			
Extension	4.15 ± 5.89	0.04 ± 3.01	.001
Flexion	101.80 ± 16.74	117.56 ± 15.61	.001
Final follow‐up			
Extension	2.77 ± 7.45	0.53 ± 2.54	.058
Flexion	87.54 ± 17.73	109.94 ± 13.91	.001

LOA, lysis of adhesions; ROM, range of motion; SD, standard deviation; TKA, total knee arthroplasty.

### Intraoperative ROM Gains

Intraoperative ROM gains were comparable between surgeons with arthroscopic training and those without, and no statistical differences were observed. Among patients treated by arthroscopically trained surgeons, the mean flexion gain during the procedure was 35.2° ±11.4°, whereas those treated by nonarthroscopically trained surgeons had a mean flexion gain of 31.1° ± 12.6° (*P* = .130). Similarly, mean extension changes, measured as the reduction of a flexion contracture toward full extension, improved by 2.43° ± 3.8° in the arthroscopically trained group and 3.91° ± 4.2° in the nonarthroscopically trained group (*P* = .079) (Table 3).

**TABLE 3:** Functional Outcomes and Complications Stratified by Surgeon Arthroscopic Training Status

Outcome	Nonarthroscopy Trained (n = 48)	Arthroscopy Trained (n = 46)	*P* Value
Intraoperative ROM gains			
Flexion gain, ° (mean ± SD)	31.1 ± 12.6	35.2 ± 11.4	.130
Extension change, ° (mean ± SD)∗	3.91 ± 4.2	2.43 ± 3.8	.079
Postoperative ROM retention			
Flexion retained, % of intraop gain	49%	86%	<.001
Flexion loss from intraop peak, ° (mean ± SD)	15.98 ± 14.2	4.85 ± 7.6	.006
Extension change postop vs intraop, ° (mean ± SD)	−1.58 ± 3.4†	0.50 ± 2.9	.036
Final ROM relative to pre‐TKA baseline			
Net flexion change, ° (mean ± SD)	−21.76 ± 18.9†	1.66 ± 8.1	<.001
Net extension change, ° (mean ± SD)	0.00 ± 3.1	1.02 ± 2.7	.277
Complications			
Revision TKA, n (%)	6 (12.5%)	2 (4.3%)	.140
Persistent stiffness	4	1	‐
Aseptic loosening	2	1	‐
Periprosthetic joint infection, n (%)	1 (2.1%)	0 (0%)	‐

intraop, intraoperative; postop, postoperative; ROM, range of motion; SD, standard deviation; TKA, total knee arthroplasty.

∗ Change in extension reflects improvements toward 0°.

† Negative values indicated loss in flexion or extension.

### Postoperative ROM Retention

At the latest follow‐up, patients treated by arthroscopically trained surgeons preserved 86% of their intraoperative flexion gains compared with 49% in the nonarthroscopic group (*P* < .001). The mean flexion loss from the intraoperative peak was 4.85° ± 7.6° in the arthroscopically trained group compared with 15.98° ±14.2° in the nonarthroscopic group (*P* = .006). Additionally, extension continued to improve postoperatively in the arthroscopically trained cohort with a mean change of 0.50° ± 2.9°, although a mean loss of 1.58° ± 3.4° was seen in the nonarthroscopic group (*P* = .036) (Table 3).

### Final Functional ROM Relative to Pre‐TKA Baseline

When referenced to each patient's documented ROM prior to their index primary TKA, the arthroscopically trained group showed a small but positive net improvement of 1.66° ± 8.1°, whereas the nonarthroscopically trained cohort experienced a significant mean loss of 21.76° ± 18.9° (*P* < .001). For extension, there was no statistically significant difference between the 2 groups, with a mean improvement of 1.02° ± 2.7° in the arthroscopic group compared with 0.00° ± 3.1° in the nonarthroscopic group (*P* = .277) (Table 3).

### Complications and Reoperations

Overall, complication incidence was low. A total of 8 knees (8.5%) required revision TKA across the whole cohort, with 5 cases attributed to persistent stiffness and 3 to aseptic loosening. Of these, 6 revisions occurred in the nonarthroscopically trained group and 2 in the arthroscopically trained group (12.5% vs 4.3%, *P* = .140). There was 1 case of periprosthetic joint infection (1.1%), which developed almost 2 years after LOA in a case performed by a nonarthroscopically trained surgeon (Table 3). Kaplan‐Meier survival analysis showed no statistically significant difference in implant revision‐free survival between the 2 groups (log‐rank *P* = .390) (Figure 2).

**FIGURE 2:**
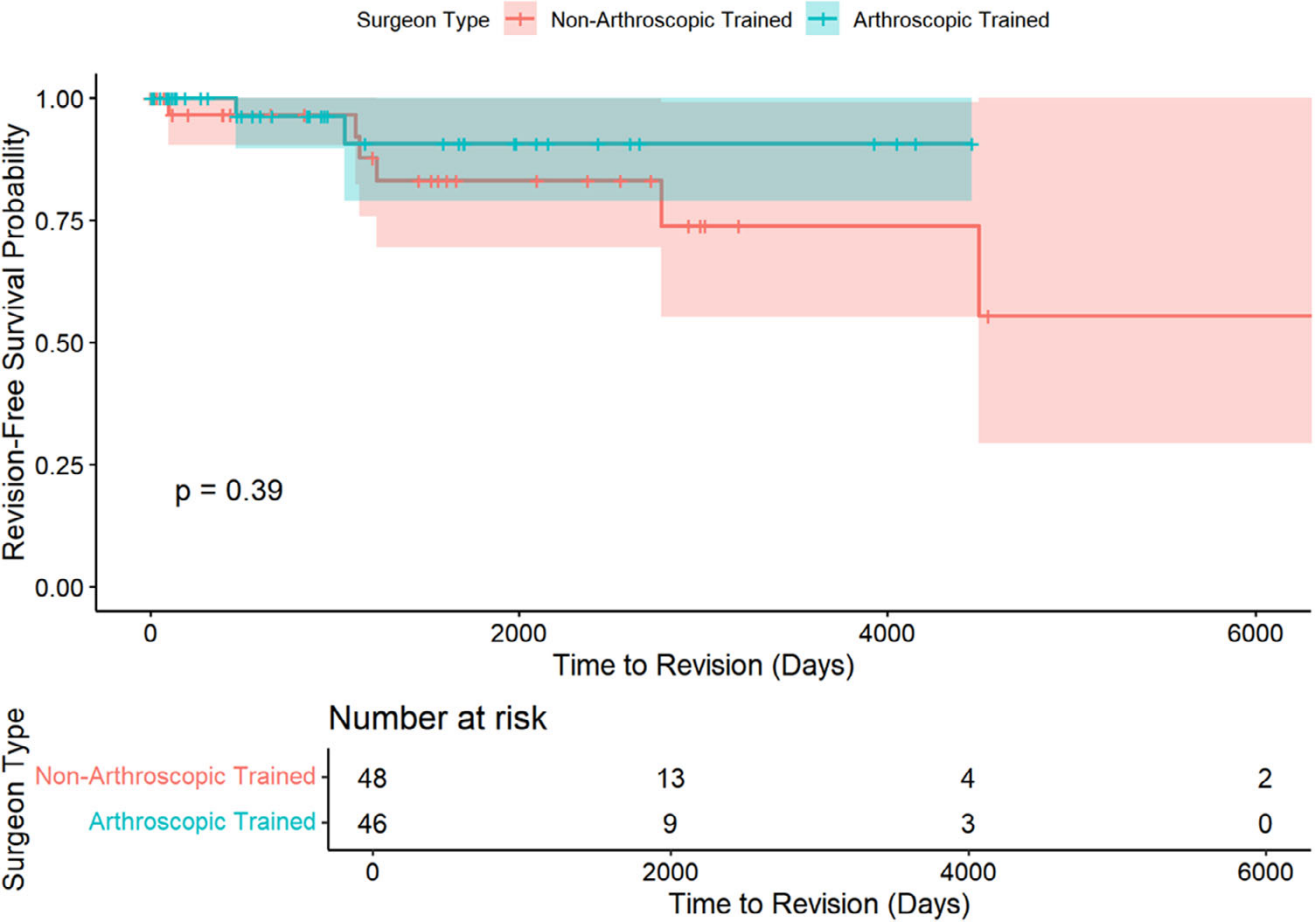
Kaplan‐Meier curve depicting revision‐free survival stratified by surgeon arthroscopic training status. Kaplan‐Meier analysis of time to revision TKA among patients treated by arthroscopically trained versus nonarthroscopically trained surgeons. The difference in revision‐free survival between the groups was not statistically significant (log‐rank *P* = .39). Shaded areas represent 95% confidence intervals. Tick marks indicate censored cases. The number at risk at various time points is displayed below the x‐axis. (TKA, total knee arthroplasty.)

## DISCUSSION

The most important finding of this study is that LOA performed by arthroscopic fellowship‐trained surgeons results in significantly superior long‐term retention of ROM compared with nonarthroscopically trained surgeons. Although intraoperative flexion gains were statistically equivalent between groups, patients treated by arthroscopically trained surgeons retained 86% of their gains at final follow‐up, whereas the nonarthroscopic group retained only 49%. Furthermore, the arthroscopic cohort achieved a net improvement compared with their preprimary TKA baseline, whereas the nonarthroscopic group experienced a significant net loss of motion relative to their original preoperative status.

Postoperative stiffness affects 1% to 6% of contemporary primary TKAs and remains one of the leading indications for early revision, with earlier studies reporting rates up to 13%.^12^, ^13^ Arthrofibrosis is therefore not a rare complication, and its impact on quality of life, health care use, and cumulative revision risk continues to drive investigations on modifiable risk factors and optimal treatment algorithms. Early single‐center case series in the 1990s laid the groundwork for LOA as a safe technique to address post‐TKA arthrofibrosis. Diduch et al. reported 26° of mean flexion gain in 8 stiff knees,^14^ whereas Court et al. noted a 31° improvement at a median 6‐month interval from the index arthroplasty with an equally benign complication profile.^15^ Small European cohorts by Djian et al. (n = 6) and Klinger et al. (n = 27) showed 30° of additional motion and meaningful Knee Society Score gains, with just 1 periprosthetic joint infection in the latter series.^16^, ^17^ Through the 2000s, larger case series consolidated these findings. Jerosch et al. (32 knees) and Arbuthnot et al. (22 knees) documented 22° to 24° flexion increases and durable functional benefits without major complications.^18^, ^19^ Hegazy et al.'s cohort (8 knees) emphasized parallel improvements in pain and function, whereas Schwarzkopf et al. (19 knees) highlighted that patients with higher body mass index paradoxically achieved greater gains, again with no infections noted.^20^, ^21^ A 2022 systematic review synthesizing 8 studies (240 knees) calculated a pooled 41.6° mean ROM improvement with only 1 minor complication (0.4%); however, the durability of those gains varied widely because up to one‐third of patients lost >10° of flexion within 2 years.^8^ In the present study, both groups achieved intraoperative flexion gains (35.2° and 31.1°) consistent with these published benchmarks, confirming that adhesion release produces similar immediate results regardless of training background. However, the nonarthroscopic group's mean flexion loss of 15.98° from intraoperative peak aligns with the pattern of postoperative deterioration described in this literature, whereas the arthroscopic group's loss of only 4.85° represents a meaningfully more durable result. This suggests that surgeon arthroscopic expertise may be a previously unrecognized and important variable to the difference in ROM retention reported across studies.

More recent large database investigations have, however, tempered this optimism. A multicenter cohort of 425 LOAs found complication and reoperation rates significantly higher than early or late MUA despite equivalent ROM gains.^22^ TriNetX data on 489 early LOAs showed greater short‐ and long‐term morbidity compared with MUA, and a PearlDiver study of 2997 LOAs linked the procedure to elevated periprosthetic joint infection and revision TKA risk relative to matched controls.^23^, ^24^ Conversely, a recent single‐practice series spanning 2012 to 2022 (103 LOAs) reported a 60° total‐arc gain at a mean follow‐up of 1.7 years but still observed a 13.6% revision rate and identified male sex, higher body mass index, and worse pre‐LOA flexion as risk factors for subsequent revision.^2^ In our cohort, the overall revision rate was 8.5%, with the nonarthroscopic group's rate (12.5%) aligning more closely with the higher rates reported in these database studies. These findings raise the possibility that the mixed outcomes reported in the broader LOA literature may be partially attributable to unexamined variation in surgeon arthroscopic expertise.

Overall, the literature has shown favorable evidence regarding LOA in TKA arthrofibrosis, yet mixed data still exists, which is why it is important to further explore variables that could influence LOA success such as surgeon‐related factors. In the present cohort, long‐term ROM retention in fellowship‐trained cases (86% of the intraoperative gain) represents a high rate of retention consistent with or exceeding those reported in the literature, supporting the premise that how the arthroscopy is performed, rather than simply what is achieved intraoperatively, can dictate sustained benefit. Surgeon‐specific factors are increasingly recognized as quality metrics across orthopaedic surgery. Nationwide database analyses of 1489 surgeons found that sports medicine fellowship training and higher arthroscopic volume reduced 2‐year reoperation and 90‐day readmission after rotator cuff repair.^10^ In arthroplasty, operating at a lower‐volume center has been associated with nearly double the risk of revision surgery compared with high‐volume counterparts.^25^ Our study extends these concepts to a prosthetic joint arthroscopy: despite similar intraoperative ROM changes immediately after LOA, arthroscopy‐trained surgeons had more durable ROM improvements and trended toward fewer revision rates.

Several mechanisms may underlie the observed advantage associated with arthroscopically trained surgeons. Comprehensive 3‐compartment debridement is a cornerstone of effective LOA, with surgical technique series emphasizing the thorough removal of adhesions across the suprapatellar pouch, the intercondylar notch, the pretibial recess, and the medial and lateral gutters to minimize residual scar tissue.^26^, ^27^ Fellowship training typically includes high procedural volume and direct mentorship in complex arthroscopic techniques, which likely enhances a surgeon's ability to perform more comprehensive and complete debridement. Instrument efficiency and tissue preservation may also play a role. Biologically, arthrofibrotic capsules are enriched with actin alpha 2, smooth muscle, collagen type I alpha 1 chain, and other transforming growth factor beta‐driven extracellular matrix transcripts, and profibrotic cytokine activation perpetuates collagen deposition and resistance to apoptosis, making any incremental surgical trauma a potential catalyst for recurrent contracture.^28^, ^29^ With iatrogenic injuries of different severities being common, surgeon familiarity with arthroscopic shavers and radio frequency probes in tight prosthetic envelopes likely shortens operative times and minimizes synovial injury that could trigger further inflammatory response and refibrosis.^30^, ^31^

Our findings also refine the place of LOA within the treatment algorithm. Early MUA remains the least invasive and most cost‐effective option.^32^ For patients presenting later, or after a failed MUA, LOA performed by arthroscopy surgeons offers a meaningful chance of a more sustained functional recovery. Future work should incorporate prospective multicenter registries with propensity matching for surgeon volume and experience, patient‐reported outcomes, and long‐term cost analyses. Adjunctive pharmacologic strategies that target the transforming growth factor beta and myofibroblast axis could be tested alongside technically optimized LOA to further improve durability.^33^, ^34^

### Limitations

Several limitations need to be considered. Owing to the retrospective nature of the study, causal inferences are limited, and all data were dependent on the accuracy and completeness of existing documentation. Furthermore, ROM measurements were performed by multiple surgeons and clinical care team members over the 35‐year study period without a standardized measurement protocol. Although goniometers are standard instruments in our clinical practice, we cannot verify their consistent use across all historical encounters. This interobserver and interinstrument variability may have introduced measurement error, although any such error would be expected to affect both groups equally given the retrospective, nonrandomized study design. Given the sample size and low complication event rate, analyses were restricted to univariate comparisons to avoid overfitting. Additionally, patient‐reported outcomes were not consistently available across the study period, limiting our ability to correlate ROM gains with subjective functional improvements. The cohort spans 35 years, during which implant designs, surgical technology and approaches, anesthetic regimens, and rehabilitation protocols evolved substantially. Surgeon‐level variables other than fellowship training (i.e., annual case volume, career stage, and era‐specific technique) were not controlled. High‐volume arthroscopy surgeons might achieve better results irrespective of fellowship status, and some arthroplasty surgeons may have acquired substantial arthroscopic proficiency through experience alone. In addition, consistency of follow‐up with physical therapy could not be fully documented because of unavailable physical therapy documentation from outside institutions. Additionally, sex‐disaggregated outcome analysis was not performed because of insufficient subgroup sample sizes when stratified by both patient sex and surgeon training type, which would have limited statistical power. Future larger studies should examine whether patient sex modifies the relation between surgeon training and ROM outcomes after LOA.

## CONCLUSIONS

LOA performed by arthroscopic fellowship‐trained surgeons results in significantly better retention of postoperative ROM than nonfellowship‐trained surgeons when used for arthrofibrosis after TKA.

## DISCLOSURES

The authors (G.B., A.C‐B., C.N., E.L., M.H., C.J.O., A.C., J.B.) declare that they have no known competing financial interests or personal relationships that could have appeared to influence the work reported in this article.
